# Melatonin as an Antioxidant and Immunomodulator in Atopic Dermatitis—A New Look on an Old Story: A Review

**DOI:** 10.3390/antiox10081179

**Published:** 2021-07-24

**Authors:** Andrzej Kazimierz Jaworek, Jacek Cezary Szepietowski, Przemysław Hałubiec, Anna Wojas-Pelc, Jolanta Jaworek

**Affiliations:** 1 Department of Dermatology, Jagiellonian University Medical College, 31-501 Cracow, Poland; anna.wojas-pelc@uj.edu.pl; 2 Department of Dermatology, Venereology and Allergology, Wroclaw Medical University, 50-368 Wroclaw, Poland; jacek.szepietowski@umed.wroc.pl; 3 Student Scientific Group, Department of Dermatology, Jagiellonian University Medical College, 31-501 Cracow, Poland; przemyslawhalubiec@gmail.com; 4 Department of Medical Physiology, Faculty of Health Sciences, Jagiellonian University Medical College, 31-126 Cracow, Poland; jolanta.jaworek@uj.edu.pl

**Keywords:** atopic dermatitis, melatonin, sleep disturbances

## Abstract

Atopic dermatitis (AD) is common inflammatory dermatosis, typically with chronic and recurrent course, which significantly reduces the quality of life. Sleep disturbances are considered to be remarkably burdensome ailments in patients with AD, and are routinely included during assessment of disease severity. Therefore, endogenous substances engaged in the control of circadian rhythms might be important in pathogenesis of AD and, possibly, be used as biomarkers of disease severity or even in development of novel therapies. Melatonin (MT), the indoleamine produced by pineal gland (but also by multiple other tissues, including skin), plays a pivotal role in maintaining the sleep/wake homeostasis. Additionally, it possesses strong antioxidant and anti-inflammatory properties, which might directly link chronic skin inflammation and sleep abnormalities characteristic of AD. The objective of this work is to systematically present and summarize the results of studies (both experimental and clinical) that investigated the role of MT in the AD, with a focus on the antioxidant and immunomodulatory effects of MT.

## 1. Introduction

Atopic dermatitis (AD, commonly referred to as eczema) is considered to be the most frequent inflammatory skin disease, with global prevalence of 15 to 20% among children and up to 10% in adults [1,2,3]. The total burden of this dermatosis (i.e., disability adjusted life-years) is the largest of all skin diseases [4]. Both the incidence and the severity of AD are influenced by the environmental factors such as air pollutants or animal-derived allergens [5].

The clinical presentation of the patient with AD typically involves chronic and relapsing erythematous patches with exudation, often accompanied by papules, oedema and crusting with characteristic, age-dependent, anatomical distribution [6,7,8]. A foundation for theses abnormalities is persistent inflammatory state with skin xerosis. With time, hypo- and hyperpigmentation occurs, replacing the healing lesions. Likewise, chronic scratching results in lichenification and fissuring. In children (the onset is usually between 2–6 months of life) areas involved are: face and cheeks, arms and wrists, and legs. Parallel with the increasing age of the patient, skin changes tend to localize on popliteal flexures and hands, feet, neck and the periocular region. The main reported symptom, being referred to as the hallmark of the disease, is intense pruritus (leading to the itch-scratch cycle) [9,10].

The pathophysiology of AD involves the overlapping effects of impaired epidermal barrier (resulting in increased water loss and skin dryness, which could be addressed, for example, to the low expression of filaggrin) and immunological aberrations (polarization of immunological response towards T_H_2-dependent immunity, with interleukins (IL) such as IL-4, IL-13, IL-25 and IL-31 playing a prominent role, which activates Janus kinases (JAK) and triggers synthesis of IgE) [11,12]. Determination of the initial component is not straightforward, however, the division of AD into the extrinsic (initially immunological abnormalities) and intrinsic (initially abnormal skin barrier) types seems to address that problem [13].

Commonly, AD is accompanied by the other atopic diseases, i.e., associated with type 1 Gell-Coombs immediate hypersensitivity, such as: allergic asthma, allergic rhinoconjunctivitis or food allergy. The specific sequence of their development is described as the “atopic march” [14]. A higher tendency to develop skin infections (*S. aureus* is considered to be usually responsible for dysbiosis of skin microbiota observed in AD), autoimmune and rheumatologic disorders, psychiatric conditions (anxiety and depression) and, possibly, cardiovascular disorders has also been observed [15].

Sleep loss is an undisputed feature of AD, being considered during assessment of disease severity using the Scoring Atopic Dermatitis (SCORAD) scale [16]. The prevalence of sleep disturbances among AD patients was assessed as 50–80% in children and 30–90% in adults [2,17,18]. Abnormalities may be observed at any stage of sleep, such as prolonged sleep onset latency, nighttime awakenings, difficulty waking up, and serious daytime sleepiness. Data acquired by objective techniques (i.e., polysomnography (PSG) or actigraphy) indicate that sleep efficiency among AD subjects is significantly lower than in healthy controls, with reduced non-rapid eye movement (NREM) phase of sleep cycle [19]. Some additional sleep-specific ailments are observed more frequently in AD patients compared to the healthy population, including obstructive sleep apnea (OSA) and parasomnias. The quality of life (QoL) is drastically tampered by sleep disorders, resulting in overwhelming frustration and exhaustion of both patients and their families (for example sleep disorders in AD children strongly correlate with maternal depression) [20]. Although the main AD symptoms, such as excessive itch and the consequent scratching, seem to sufficiently explain the abnormal sleep pattern among patients, the observation that only about 15% of AD children were reported to be woken up by the urge to scratch, raised suspicion that actually more complex mechanisms are involved [17]. A vast variety of interleukins (particularly IL-4 and IL-31), chemokines (CXCL9 and CXCL10), neuropeptides (substance P), hormones (cortisol) and even allergen specific IgE (for example against *Dermatophagoides pteronyssinus*) were linked to abnormal sleep in AD [18]. Another molecule which provisionally plays a crucial role in sleep disturbance in AD is melatonin (MT).

Since its discovery by dermatologist Aaron Lerner in 1958, melatonin (*N*-acetyl-5-methoxytryptamine) became one of most fascinating substances [21,22]. This indoleamine was best recognized as a product of pineal gland but further studies revealed that this substance with an evolutionary conserved structure could be produced in many human tissues and in every living species. Melatonin was detected in the bacteria, fungi, plants, vertebrates and also in invertebrates. Its primary function is to serve as first-line defense against internal and environmental oxidative stressors [22,23,24,25]. Identification of MT in archaea and prokaryotes (*Cyanobacteria*) indicates that this substance was present on the Earth at the very early stage of evolution, when intensive ultra-violet (UV) radiation and high concentration of oxygen in the atmosphere posed a serious danger to living organisms. Melatonin’s ability to neutralize free radicals protected life and made the evolution of living creatures possible [22,26,27,28].

Synthesis of MT and its release from the pineal gland is driven by the light-dark cycle via the retino-hypothalamic (suprachiasmatic nucleus) axis and sympathetic nerves. Plasma levels of MT reach the highest values at night (between 1.00 and 3.00 a.m.) increasing up to 300 pg/mL, whereas light reduces MT release [29]. Because of its rhythmic fluctuations, MT is considered a main body regulator of circadian rhythms. Production of this indoleamine decreases with age, and its circadian rhythm of secretion disappears in older individuals [30].

MT has been identified in many mammalian cells and tissues such as: gastrointestinal and reproductive systems, macrophages, lymphocytes, endothelial cells, retina, salivary glands, kidneys, thyroid, pancreas, liver, spleen, airway epithelium, carotid body and brain [24,31,32,33,34,35,36,37].

MT is implicated in numerous aspects of skin homeostasis and its concentration in the skin is several times higher than in the blood [38]. Skin protection against UVB radiation is one of most known effects of melatonin, presented in numerous in vitro and in vivo studies [39,40,41,42]. MT is able to enhance the skin barrier by stimulation of keratin expression [38] and because of its effect on fibroblast, it appears as a promising agent in wound healing [43,44]. Antiaging action of MT in the skin is in line with its antioxidant/anti-inflammatory effects through inhibition of nuclear factor κB (NF-κB) and reactive oxygen species (ROS) formation, suppression of metalloproteinases (MMPs) and cyclooxygenase 2 (COX2) [45].

Skin possess a specific melatonergic system, which was evaluated by detection in the skin cells of genes and enzymes involved in MT synthesis; arylalkylamino-*N*-acetyl-serotonin-transferase (AA-NAT) and *N*-acetylserotonin *O*-methyltransferase (ASMT) [46,47]. In addition to enzymatic production, MT synthesis in the skin might be triggered by UVA-induced free radical oxidation of L-tryptophan converted to hydroxytryptophan. Among the metabolic derivatives of melatonin, there are: 6-hydroxymelatonin (6-OHM), 4-hydroxymelatonin (4-OHM), *N*^1^-acetyl-*N*^1^-formyl-5-metoxykynuramine (AFMK), and *N*^1^-acetyl-5-methoxy-kynuramine (AMK). All the above metabolites are strong antioxidants, in the same way as their maternal molecule [48,49].

MT, as it is highly lipophilic, easily penetrates through cell membranes, to directly affect intracellular organelles. Along with this direct action of MT its biological effects are also mediated by its receptors MT1R, MT2R, MT3R [50,51,52]. MT membrane receptors MT1R and MT2R belong to G-protein-coupled receptors. Receptor MT3 is the enzyme quinone reductase 2 (QR2), involved in antioxidant action of MT [53]. Both receptors MT1R and MT2R have been identified in mammalian skin, eccrine glands, and blood vessels; however, MT1R is predominant and it was found also in the differentiating layer of epidermis [54].

An additional group of MT receptors are orphan nuclear receptors (retinoid orphan receptor, ROR), which have been identified in skin cells [52,55]. The implication of these receptors on the effects of MT is the matter of discussion. Some authors questioned their role as MT receptors, explaining that natural ligands for these receptors are sterols, which are structurally different from MT [50]. Experimental studies by Dai et al. demonstrated that RORα/β could be involved in pathogenesis of atopic dermatitis [56].

MT is generally accepted as versatile protector against oxidative damage. Mitochondria are main cellular source of ROS and reactive nitrogen species (RNS). MT controls the generation of these free radicals through modulation of mitochondrial genes expression and improves activity of mitochondrial respiratory chain [22,28,51,52,57,58,59,60]. It should be emphasized that MT is also synthesized in these organelles by aforementioned enzymes, AA-NAT and ASMT [61]. Actually, mitochondrial concentration of MT is virtually independent of the pineal gland synthesis (although mitochondria are capable of up-taking MT by transporters PEPT1/2) and mitochondria-derived MT is generally not released into systemic circulation. Surprisingly, mitochondrial MT constitutes more than 95% of total MT production [60]. On the other hand, MT plays a pivotal role in the regulation of mitochondrial biogenesis and in maintaining their homeostasis (referred to as mitoprotection), which involves, for example, reduced cytochrome c release and lowered Ca^2+^ overload [62,63].

The antioxidant activity of MT in mitochondria involves direct scavenging of ROS, as well as activation of sirtuin 3, which deacetylates (therefore, activates) mitochondrial superoxide dismutase 2 (SOD2) [61]. As the free radicals acts only over the short distance from their site of origin (i.e., their half-life time is approximately ~10^−9^ s, as is the case for HO^•^), only antioxidants present in the same cell’s compartment can effectively quench them. In mitochondria, MT seems to be the main executor of this role [60]. MT is able to protect mitochondrial membrane from the peroxidation of cardiolipin and formation of mitochondrial permeability transition pores (mPTPs), to maintain the mitochondrial membrane potential, formation of adenosine triphosphate (ATP), and prevent cells form apoptosis [60,64,65]. That activity is supported by the activation of MT1R receptors located on the outer mitochondrial membrane.

The complex activity of MT in mitochondria results in alleviation of the cytokine storm (for example through repression of mTOR and HIF-1α, and inhibition of oxidative glycolysis), synergistically with MT produced peripherally by pineal gland [63]. It is interesting that MT could also induce mPTP and is able to activate the apoptotic process in cancer cell lines [66]. Reiter et al. [67] pointed out that the circadian rhythm of MT synthesis (present also in mitochondria) contributes significantly to difference in the metabolic pattern between the day and night, with oxidative phosphorylation augmented by MT.

Protective effects of MT on cellular structures, mainly on deoxyribonucleic acid (DNA), are attributed to its potent antioxidant ability [68]. MT exerts its antioxidant action in multiple ways:

1. MT, by donating an electron, acts a direct scavenger of reactive species. In this way it neutralizes variety of free radicals such as HO^•^, alkyl radicals (RO^•^), peroxyradicals (ROO^•^) and ^•^NO, as well as non-radical oxidants: singlet oxygen (^1^O_2_), hydrogen peroxide (H_2_O_2_) and peroxynitrate (ONOO^−^).

2. MT indirect effect is related to the stimulation of the antioxidant enzymes such as: superoxide dismutase, catalase (CAT), glutathione peroxidase (GPx), glutathione reductase (GPd), glutathione S-transferase (GTS), heme oxygenase 1 (HO-1), γ-glutamyl-cysteine synthase (γ-GCS). This indirect effect is dependent on the activation of nuclear erythroid 2-related factor (Nrf2) by MT and was demonstrated in many cells, including keratinocytes [69,70,71,72,73,74].

3. MT was demonstrated to inhibit pro-oxidative enzyme xanthine oxidase (XO), which is known to generate radicals in tissues exposed to microwave radiation [75,76].

4. MT is able to accelerate the repair of ROS-induced DNA lesion. It enhances the expression of DNA repairing genes, such as 8-oxoguanidine glycosylase (OGG1), and affects the mechanisms involved in DNA repair pathways [77,78].

Antioxidant effect MT is accompanied by its anti-inflammatory activity. MT reduces the inflammatory process through widespread mechanisms involving modulation of intracellular signaling pathways, inhibition of proinflammatory NF-κB, and activation of antioxidant Nrf2 pathways. Experimental studies demonstrated that MT reduces generation of proinflammatory cytokines and enzymes such as: inducible nitric oxide synthase (iNOS), COX2, tumor necrosis factor α (TNFα), interleukin 1β (IL-1β), Il-4, IL-6, IL-13, or IL-18 [79,80,81]. Recent studies have presented MT’s anti-inflammatory action related to the suppression of inflammasome NLRP3, which amplifies the NF-κB-mediated inflammatory response. Inhibition of NLRP3 by MT prevented from the activation of pro-L-1β and pro-IL-18, thus blocking the initiation of proinflammatory cytokine cascade [82,83,84,85].

**Figure 1:**
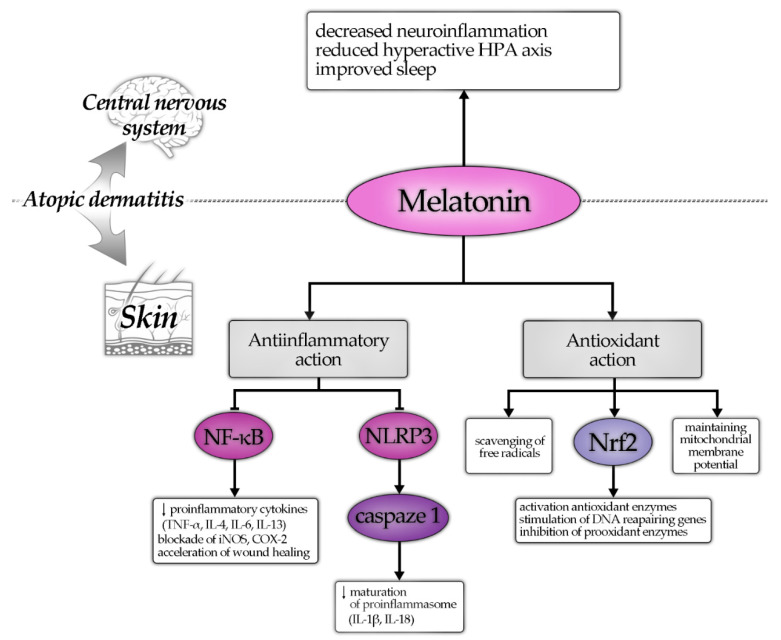
Protective effects of melatonin on atopic dermatitis—hypothetic mechanisms of action. Note: the arrow (↓) means, that the decrease in given entity is observed.

The above discussed mechanisms and effects of MT are summarized in Figure 1.

It should be mentioned that MT was earlier presented in in vitro studies as immunomodulatory molecule. Studies on monocytes and T cells demonstrated that MT upregulated expression of proinflammatory cytokines, in particular IL-17, which subsequently initiates release of other interleukins (TNFα, IL-1β, IL-6) [83,86,87].

## 2. Materials and Methods

This review was based on the analysis of the PubMed database [88]. Articles were searched through Medical Subject Headings (MeSH) thesaurus, looking for the publications related to MT and AD. The key terms “melatonin” AND (“atopic dermatitis”, OR “atopic eczema”, OR “eczema”) were used for main search.

The initial search on MT and AD selected 28 articles, 31 publications on MT and atopic eczema and 12 articles on MT and eczema. Selected articles were published from 1988 to 2021. Most of the articles from the above three searches were the same papers. The abstracts of articles in which title suggested the relation between MT and AD were selected and read. Publications identified from the abstract as irrelevant to the topic, review papers, or these not written in English were excluded. Sole abstracts without any access to the article, were also excluded, except one.

Articles selected for analysis were carefully read, and their references were examined to identify the publications, that could be included in the review.

## 3. Results

Articles searched from PubMed regarding melatonin MT and AD were sparse. For final analysis, 11 original papers and a single abstract were selected. Among these papers, there were 8 that presented results of clinical studies and 3 describing experimental studies on animals. A single abstract was included into analysis, because it was the first published information on MT and AD that we were able to find, and because of few publications on the above topic.

The first report on MT and AD that we were able to find comes from 1988. Subsequent clinical papers were published: 3 in 2007, 1 in 2014, 2 in 2016, 1 in 2018, and the last one in 2020. Three experimental studies on NC/Nga atopic such as mice and melatonin were performed in 2009, 2017 and 2018.

Two clinical studies employed adult AD patients, while the rest of papers presented observations on atopic children and adolescents. Study groups consisted of 20–75 persons. Most studies presented results obtained from an Asian population: two were from China, two from Japan, one from India, Turkey and Iran. Two additional studies originated from Europe (Germany and Spain).

Table 1, Table 2 and Table 3 present the main data that form the results of this study.

### 3.1. MT Secretion and Blood Level in Patients with AD

Studies on the melatonin secretion and its blood concentration in AD patients presented opposite results.

The first report suggesting possible implication of MT in pathophysiology of AD was reported by Schwarz et al. [89] in 1988. Their study evaluated serum MT concentrations in adult patients with AD. This study involved a limited number of patients (18 persons). In six of the AD patients, the serum level of MT was lower than in control subjects and the circadian rhythm of MT was abolished. An additional eight patients presented a reduced nocturnal peak of serum MT. Only four patients presented normal secretion pattern of MT. Authors related abnormalities of MT secretion to the low activity of the sympathetic nervous system.

This observation was supported by two original studies concerning MT secretion by mammary and salivary glands. In both the above secretions, the MT concentrations originating from patients suffering from AD were decreased compared to the healthy subjects [90,91]. One of these studies involved 48 lactating women with mild AD and the same number of healthy mothers. MT was measured in their milk under basal conditions and following nonstandard stimulation, which was the presentation of 87 min humorous films with Charlie Chaplin. The control women observed weather information. MT was measured every 2 h, during the night. The duration of the experiment was 2 weeks. MT secretion to the milk had a peak at 2.00 a.m. and reached 10.7 pg/mL in AD mothers and 15.6 pg/mL in healthy women. Concentration of MT in the milk of all mothers viewing humorous films was significantly elevated, and peak value in AD group was similar to control (19.9 pg/mL vs. 20.8 pg/mL) [90]. The author did not explain the mechanism of this phenomenon; however, he suggested that feeding babies with milk with a higher concentration of MT may reduce the allergic response in infants with AD.

In the next study, the author, using the same model of experiment as that used for the lactating women (viewing humorous films), investigated MT concentration on saliva. The study group enrolled 40 AD children with mild and moderate severity of this disease. The author observed lower concentration of nocturnal (measured at 2.00 a.m.) MT in saliva of patients with AD (26.5 pg/mL), as compared to healthy control (58.6 pg/mL). Additionally, stimulated secretion of MT was diminished in atopic children (51.9 pg/mL vs. 66.7 pg/mL) [91].

Decreased serum melatonin MT was also reported in a subsequent report, which was carried out on 40 atopic children and adolescents. Reduction in daily MT serum concentration was demonstrated in the AD group, compared to healthy control (11.7 pg/mL vs. 30.5 pg/mL). Nocturnal peak of MT was lower in AD patients (34.5 pg/mL), than in the compared healthy group (43.5 pg/mL), but the difference was not significant [92].

More recent studies presented opposite results. Nocturnal MT secretion, assessed as urinary concentration of 6-sulfatoxymelatonin at night, was higher in children with moderate AD, compared to in analogous healthy group [93]. Additionally, two successive clinical trials revealed that serum MT measured in the morning in AD children was increased, compared to the control patients [94,95]. The serum MT levels achieved in AD children was 1.6 pg/mL, whereas in healthy control was 0.92 pg/mL [95]. In the second study, MT serum concentration in atopic patients reached 2.1 pg/mL, which was significantly higher than in control—1.6 pg/mL [94]. In one of these studies, the correlation between blood MT and severity of disease was observed, with lower concentration of MT found in persons suffering from more severe AD [94]. However, in another recent study, such a relationship has not been found [95].

### 3.2. Oxidative Stress Markers, Melatonin and Severity of Atopic Dermatitis

The oxidative stress indicators measured in AD patients included: MDA, NO and antioxidant enzymes: SOD and GPx, and a positive correlation was found between NO/melatonin and malondialdehyde/melatonin ratios (r = 0.511, *p* < 0.0001) [94,95]. All markers were measured in the patient’s serum. MDA was measured in two studies and the results were opposite. In one of these studies, the values of MDA were low, and MDA in AD children was significantly higher (0.12 nmol/L) as compared to healthy individuals (0.06 nmol/L) [95]. The authors reported no correlation between MDA value and severity of disease. In the next study, MDA in AD children was not significantly different from healthy control and reached 1.28 nmol/L in investigated AD patients vs. 1.30 nmol/L in control. On the contrary, serum NO level was decreased in AD patients (28.90 nmol/L) when compared to control value (33.55 nmol/L). Additionally, NO/MT ratio was markedly lower in study group (2.26) than in control (4.97) [94].

Antioxidant enzymes GPx and SOD were higher in atopic children (246.52 pg/mL, and 9.48 pg/mL, respectively) than in the control group (209.54 pg/mL and 7.86 pg/mL, respectively), but the differences were not statistically significant [95].

In the mice model, DNCB-induced neuroinflammation treatment with MT reduced both hippocampal COX2 (from 116.4% to 105.4% of control) and iNOS (from 124.6% to 107.4% of control) [100].

### 3.3. Melatonin and Sleep Quality in Patients with Atopic Dermatitis

It is commonly accepted that MT facilitates a transition to sleep in humans. The relationship between MT and sleep was investigated in four analyzed studies on MT in AD patients. The obtained results were not univocal, and differences related to the details of presented data have been found. In three of publications, higher secretion of MT and its increased serum concentration was positively correlated to the sleep quality [91,93,96]. In AD children with low serum level of MT, disturbances of sleep such as decreased non-rapid eye movement (NREM) phase, and longer onset of sleep latency were recorded. It was perceived that a higher SCORAD index was significantly correlated with poorer sleep, shorter total sleep time and more sleep fragmentation. Authors concluded that higher SCORAD predicted poor sleep efficiency [93]. However, in a later study of the same group of authors, the association between improvement of SCORAD index and sleep has not been found [96]. In their recent study, Ardakani and his group demonstrated the lack of correlation between serum MT and improvement of sleep-onset latency in young patients with AD, but it should be highlighted that MT alleviated AD (improved SCORAD) independent of the disease’s negative effect on sleep [97].

In one study, β-endorphin in serum was measured and was found to be lower in atopic children (26.24 pg/mL) than in control (45.33 pg/mL) [92].

### 3.4. Effect of MT Supplementation on Atopic Dermatitis

Two separate studies evaluated the effect of MT supplementation on the severity of AD and sleep disturbances. In both studies, MT was given to the patients before sleep time every day for several weeks. In the study by Chang et al. [96] MT at dose of 3 mg/day was applied for 4 weeks, which produced a significant increase in the nocturnal urinary MT level. In the second study, carried out by Ardakani at al. [97], the dose of MT was doubled (6 mg/day) and such treatment of AD children was continued for 6 weeks. MT application resulted in a significant reduction in SCORAD index in both studies. In the first of these studies, the reduction in sleep-onset latency was noted, yet this did not correlate with attenuation of disease severity expressed as SCORAD [96]. Supplementation with MT did not significantly affect pruritus score, sleep fragmentation, mobility in sleep, total sleep time and CRP value in both studies.

Opposite effect of MT supplementation on serum IgE was observed. In one study serum IgE concentration in patients with AD was not affected by MT, despite of reduced SCORAD [96]. In another study, serum IgE was reduced [97].

### 3.5. Results of Experimental Studies on the Effect of MT on AD

NC/Nga mice are useful models of human AD, as the inflammatory response of their skin resembles that seen in human with atopic skin inflammation. For the experiments, animals are sensitized with 2,3-dinitrofluorobenzene (DNFB), or with 2,3-dinitrochlorobenzene (DNCB) followed by subsequent application of one of these substances.

Systemic application of MT (10 or 20 mg/kg per day) for 2 or more weeks significantly decreased skin lesions and reduced serum IgE [98,99]. In experiments in vitro with CD4^+^ cells isolated from lymph nodes of mice treated with MT, the production of IL-4 and INF-γ was diminished compared with production of these interleukins secreted by CD4^+^ cells obtained from control, untreated with MT mice [98].

Two experimental studies investigated the effect of prolonged (6 weeks) MT treatment on the stress, neuroinflammation and neuronal damage in NC/Nga-atopic-like mouse model. In these animals, treatment with DNFB increased activity of HPA axis, that was evidenced by the high serum concentrations of CRH, POMC, ACTH and cortisol. Levels of the above markers of HPA axis were also augmented in hypothalamus and hippocampus of NC/Nga atopic mice. In the brain of these animals increased expression and protein signals for iNOS, COX2, IL-1α, INF-α and amplification of microglial activity were detected. Expression signals and proteins of MT synthesis and MT1R in the skin and in the hypothalamus were reduced. MT administration to NC/Nga atopic mice suppressed hyperactive HPA axis, restored the level of MT and its receptor in the brain and skin, and reduced neuroinflammation [99].

In another study by the same group of authors, the increased activity of the HPA axis in NC/Nga was assessed by measurement of catecholamines synthesis. This was evidenced by high activity of enzymes participating in this process—dopamine β-hydroxylase and tyrosine hydroxylase—and by increased concentrations of noradrenaline and dopamine in the different areas of brain. Such changes resulted in ADHD. Treatment with MT reversed the above abnormalities in the levels of neuromediators in the brain and inhibited ADHD behavior [100].

## 4. Discussion

The results of our study show that implication of MT in the pathogenesis of AD is still unexplained. One of reasons is that publications concerning this subject are sparse. We were able to find only eight clinical studies and three experimental papers from between 1988 and 2020. In addition, the data from clinical observations are sometimes incoherent.

Measurements of MT blood concentration in patients with AD presented contrasting results. In some earlier published papers, authors have shown that serum MT was significantly decreased in AD patients, comparing to the healthy subjects [89,92]. Additionally, secretion of this indoleamine into saliva and into breast milk of patients with AD reached lower values, than in the control [90,91].

On the contrary, studies performed during the last 5–6 years demonstrated that MT concentrations in serum and in urine were higher than those observed in the control groups [93,94,95]. These discrepancies could be partially related to the differences between groups of patients participating in the particular studies, to the samples collection and methods of melatonin measurement.

Patients with AD included into study group were characterized by moderate or mild SCORAD, but sometimes children with mild, moderate, and severe AD were located in the same study group [91]. Participants in one group were small children and adolescents, even though MT secretion changes with age, and the puberty stage is characterized by a decline in MT secretion [101]. Additionally, in some of studies the number of patients was limited to 20–30 persons [99,100]. Such limited study populations could produce difficulties with interpretation of results.

Samples were collected during the night, when MT secretion reaches the peak [89,90,91], but also in the morning at 9:00 a.m. [94,95] or in the morning and in the evening [92]. Analyzed material originated from the blood, saliva, breast milk and urine and different methods were used to determine MT concentrations. In most of studies, MT was measured using immunoenzymatic ELISA method [90,91,93,94,95], but in one study RIA was engaged [92]. In urine samples, MT metabolite, 6-sulfatoxymelatonin was measured [93,96].

In addition to the above discrepancies, the results of studies demonstrated that serum concentration of melatonin MT was lower in patients with severe AD having high SCORAD, than in those with mild or moderate disease activity [92,94]. Perhaps in the patients with mild and moderate SCORAD MT was utilized to neutralize free radicals and because of this process, their intensity of inflammation was reduced [91]. It is likely that in addition to sleep regulation by MT, this indolamine plays an important role as an antioxidant alleviating the inflammatory changes in AD. It is noteworthy that an increase in psychic condition of AD patients (laughing, when viewing a humorous film), resulted in a significant augmentation in MT peak secretion and decrease in blood IgE [90]. In the other study, low serum MT was correlated with decreased β-endorphin secretion [92]. The release of both MT and β-endorphin are affected by adrenergic stimulation and both systems (melatonergic and opiate) are interconnected [102]. It is likely that low affinity of one of these systems depressed the other and low secretion of MT is associated with reduced opiate production and release.

It could be hypothesized that high secretion of MT and its higher serum concentration might reflect the activation of an innate defensive antioxidant system, which counteracted skin inflammatory process. This is in accordance with the decreased MDA level and augmentation (although insignificant) of antioxidant enzymes in these patients [95]. However, in the other study, high serum MT was not paralleled to the decrease in serum MDA [94].

Anti-inflammatory effects of MT occur inseparably of its antioxidant function. The aforementioned impact on MDA level, as well as reduction in NO release, possibly contribute to the alleviation of the inflammatory state [94]. Additional evidence is acquired from the experimental studies, that showed the reduction in IL-4 and IFN-γ production [98], as well as alleviated neuroinflammation (i.e., reduced level of COX2 and iNOS) [99] after supplementation with MT. These results are in line with the previous data and could be addressed to the inhibition of NF-κB and NLRP3 inflammasome. It is conceivable that MT combats inflammation systemically rather than locally (e.g., in skin and neural system), which explains the benefits of MT supplementation on both skin symptoms and sleep disturbances in patients with AD [96,97].

Four analyzed papers investigated the correlation between MT and sleep [91,93,96,97]. It was shown that lower MT concentrations were associated with poor sleep quality, whereas supplementation with adequate dose of MT improved the sleep (this improvement was observed independently from method of assessment, i.e., sleep questionnaire [97], or actigraphy [96]). Nevertheless, reductions in SCORAD and serum IgE were detected only when a higher dose of MT (6 mg/day) was given to the patients [97].

Results of experimental studies support and complete the above clinical observations. MT administration reduced inflammatory process in the skin, which was suggested by decreased SCORAD in AD patients [97] and histological skin assessment in NC/Nga atopic-like mice, decrease in serum IgE, and decline in secretion of IL-4 and INF-γ from activated CD4^+^ cells [98,99]. In addition, MT supplementation was shown in experimental studies to reduce atopic-related stress, and stress-induced neuronal damage, and reversed dysregulated catecholamines production.

## 5. Conclusions

Considering several limitations and difficulties in the interpretation of materials presented in the analyzed studies some conclusions could be made:

1. Based on previous and recent publications [103], it could be stated that AD is related to the increase in oxidative stress, and since MT is powerful antioxidant, its implication in the defense of inflammatory reaction of AD patients is very likely.
2. Lower serum MT is correlated with more severe inflammation in AD patients.
3. MT supplementation improves sleep and, with application of adequate dose, reduces SCORAD and serum IgE in AD patients.

It is worth remembering that MT derivatives share the antioxidant and anti-inflammatory effects of its maternal molecules, and AFMK, AMK and 6-OHM are even more potent antioxidants than MT itself. Generation of these particles prolongs and strengthens the effects of MT, creating MT cascade which appears one of most important mechanisms of innate immunity in various tissues [104,105,106,107].

## Figures and Tables

**Table 1:** Summary of findings from the observational human studies.

Author and Reference	Year	Patients Enrolled (*N*)	Treatment	Parameters Tested	Tissue Samples	MT Measurement	Method	Results
Schwarz et al. [89]	1988	adults with AD (18)	n/a	MT	blood	endogenous MT measured every 2 h for 24 h	not given	low serum MT in 6 patients and abolished circadian rhythm of its release; decreased nocturnal MT peak, in additional 8 AD patients; only 4 patients showed a normal pattern of MT secretion
Kimata [90]	2007	women with AD, mild SCORAD (48)	laughter, viewing a humorous film	MT, IgE	breast milk, serum	endogenous MT measured at 11.00 p.m., at 2.00 a.m., at 0.50 a.m.	ELISA (MT)	low basal MT in milk of AD patients, however stimulated MT in milk was similar to healthy control; decreased nocturnal MT peak in these patients; higher IgE in AD mothers
Kimata [91]	2007	children with AD, moderate SCORAD (24)	laughter, viewing a humorous film	MT, sleep, SCORAD	saliva	endogenous MT measured every 2 h from 10.0 p.m., to 6.00 a.m.	ELISA (MT), ELISA (INF-γ) sleep question-naire	low basal and stimulated MT in AD patients; decreased nocturnal MT peak; increased INF-γ; better sleep correlated with higher MT
Munoz-Hoyoz et al. [92]	2007	children with AD (40) -severe (20) -asymptomatic (20)	n/a	MT, β-endorphin	blood	endogenous MT measured at 9.00 a.m. and at 9.00 p.m.	RIA (MT)	low MT at 9.00 p.m.; nocturnal MT at 9.00 p.m. slightly, but non significantly lower than control; MT in asymptomatic group similar to control; β-endorphin decreased
Chang et al. [93]	2014	children and adolescents with AD, moderate SCORAD (72)	n/a	MT, sleep, IgE, pruritus, scratching movement	urine	endogenous MT measured as 6-sulfatoxymelatonin (in morning urine samples)	ELISA (MT), actigraphy, polysomnography, sleep questionnaire	nocturnal MT higher in AD patients than in controls; lower MT correlated with poor sleep efficiency, longer onset sleep latency, decreased NREM sleep, more sleep fragmentation; IgE in serum increased
Uysal et al. [94]	2016	children with AD (73) -mild (12) -moderate (22) -severe (39)	n/a	MT, NO, malondialdehyde (MDA), SCORAD	blood	endogenous MT, measured at 9.00 a.m.	ELISA (MT), ELISA (MDA), reduction method (NO)	increased MT in AD patients, comparing to control; MT lower in severe AD, than in mild; NO decreased in all AD patient; MDA similar to control
Devadasan et al. [95]	2020	children with AD (30) -mild, -moderate -severe	n/a	MT, SCORAD, MDA, SOD, GPx	blood	endogenous MT measured at 9.00 a.m.	ELISA (MT), RANDOX (SOD, GPx), TBA-TCA-HCl (MDA)	increased MT and MDA comparing to control; SOD, GPx increased, but insignificantly; none of parmeters correlated with SCORAD

**Table 2:** Summary of findings from the experimental human studies.

Author and Reference	Year	Patients Enrolled (*N*)	Experimental Treatment	Parameters Tested	Tissue Samples	Method	Results
Chang et al. [96]	2016	children and adolescent with AD, moderate SCORAD (38)	exogenous MT given 3 mg/day for 4 weeks before bedtime	MT, sleep, IgE, SCORAD	urine	ELISA (MT), actigraphy	MT application reduced SCORAD, decreased sleep onset latency; improvement of sleep was not correlated with SCORAD; serum IgE was similar to control
Ardakani et al. [97]	2018	children with AD, moderate and severe SCORAD (35)	exogenous MT given 6 mg/day for 6 weeks before bedtime	SCORAD, sleep, CRP, IgE	blood	SCORAD, sleep questionnaire	MT application reduced SCORAD, and IgE, improved total sleep scores; CRP and pruritus were similar to control

**Table 3:** Summary of findings from the experimental animal studies.

Author and Reference	Year	Experimental Models	Experimental Design	Material and Method	Parameters Tested	Results
Kim et al. [98]	2009	NC/Nga mice	sensitization with 2,3-dinitrofluorobenzene (DNFB) followed by repeated application of DNFB on the skin + MT 10, or 20 mg/day i.p. for 2 weeks	skin, blood, CD4+ cells isolated from animals, histologic analysis, ELISA kits for interleukins and IgE	skin lesions, IgE, IL-4, INF-γ	MT treatment reduced skin lesions, such as hypertrophy, hyperkeratosis and inflammatory cell infiltration, as well as serum IgE; MT treatment inhibited production of IL-4 and INF-γ by activated CD4^+^ cells
Park et al. [99]	2017	NC/Nga mice, mouse HT 22 hippocampal cell culture, rat brain primary hypothalamic neuronal cells (RPHN)	sensitization with 2,3-dinitrochlorobenzene (DNCB) followed by repeated application of DNCB on the skin + MT 20 mg/day orally with or without cortisone 20 mg/day orally for 6 weeks; incubation of cells with various concentrations of cortisol	skin, blood, brain samples, cell cultures, PCR, Western blot, immunochemistry, immunocyto-chemistry, immuno-fluorescence, comercial kits	skin lesions; scratching behavior; neuroinflammation and neuronal cells viability; IgE; MT1R, CRH, POMC, ACTH; COX2, iNOS; TNFα, IL-4, IL-1α	MT treatment reduced skin lesions, scratching behavior and serum IgE; MT treatment reversed atopic stress-induced neuronal damage; increased reduced by stress MT and MT1R in the brain and skin and supressed neuroinflammation
Park et al. [100]	2018	NC/Nga mice, SH-SY5Y human neuronal cell culture	DNCB model + MT as above, incubation of cells with various concentrations of cortisol or MT	brain tissues, human neuronal cell culture, PCR, Western blot, immunohisto-chemistry, commercial kits	MT, CRH, CRHR 1, ACTH, norepinephrine, dopamine, dopamine β-hydroxylase, tyrosine hydroxylase	MT reversed induced by AD stress-increase in norepinephrine and dopamine, and hyperactivity of hypothalamic-hypophyseal-adrenal (HPA) axis, MT corrected dysregulated dopamine and noradrenline system, which is related to attention-deficit/hyperactivity disorder (ADHD) caused by atopic mouse model
